# Synergistic treatment of osteosarcoma with biomimetic nanoparticles transporting doxorubicin and siRNA

**DOI:** 10.3389/fonc.2023.1111855

**Published:** 2023-01-23

**Authors:** Jingtong Zhao, Xupeng Mu, Xuejia Hou, Xiaowen Zhang, Ping Li, Jinlan Jiang

**Affiliations:** ^1^ Scientific Research Center, China-Japan Union Hospital of Jilin University, Changchun, Jilin, China; ^2^ Rheumatology and Immunology Department, China-Japan Union Hospital of Jilin University, Changchun, Jilin, China

**Keywords:** osteosarcoma, drug delivery, survivin, gene tharapy, tumor target

## Abstract

**Introduction:**

Osteosarcoma tumors are the most common malignant bone tumors in children and adolescents. Their treatment usually requires surgical removal of all detectable cancerous tissue and multidrug chemotherapy; however, the prognosis for patients with unresectable or recurrent osteosarcoma is unfavorable. To make chemotherapy safer and more effective for osteosarcoma patients, biomimetic nanoparticles (NPs) camouflaged by mesenchymal stem cell membranes (MSCMs) were synthesized to induce osteosarcoma cell apoptosis by co-delivering the anticancer drug doxorubicin hydrochloride(DOX) and a small interfering RNA (siRNA). Importantly, these NPs have high biocompatibility and tumor-homing ability. This study aimed to improve the efficacy of osteosarcoma therapy by using the synergistic combination of DOX and an siRNA targeting the apoptosis suppressor gene survivin.

**Methods:**

Biomimetic NPs (DOX/siSUR-PLGA@MSCM NPs) were synthesized by coloading DOX and survivin siRNA (siSUR) into poly (lactide-co-glycolide acid) (PLGA) *via* a double-emulsion solvent evaporation method. The NPs were camouflaged by MSCMs to deliver both DOX and survivin-targeting siRNA and characterized and evaluated in terms of cellular uptake, *in vitro* release, *in vitro* and *in vivo* antitumor effects, and biosafety.

**Results:**

DOX/siSUR-PLGA@MSCM NPs had good tumor-homing ability due to the MSCMs modification. The drug-laden biomimetic NPs had good antitumor effects in homozygous MG63 tumor-bearing mice due to the synergistic effect of the drug combination.

**Conclusion:**

DOX/siSUR-PLGA@MSCM NPs can show improved therapeutic effects in osteosarcoma patients due to the combination of a chemotherapeutic drug and gene therapy based on their good tumor targeting and biosafety.

## 1 Introduction

Osteosarcoma is a highly malignant bone tumor that occurs primarily in the long bones, and its peak incidence is during adolescence. Because of its high rate of systemic dissemination, conservative surgery and local surgical treatment are often accompanied by a high risk of recurrence and metastasis. Among patients with metastatic tumors, the vast majority develop pulmonary metastases and distal bone metastases (1–3).

Before 1970, osteosarcoma could only be treated by amputation, with a poor postoperative prognosis and a low survival rate. With the development of modern medical technology, neoadjuvant therapy, induction therapy, and consolidation therapy, the treatment of patients with osteosarcoma gradually changed to limb-preserving surgical chemotherapy and reconstruction (4, 5). However, there is still a lack of new effective approaches for patients with osteosarcoma who cannot undergo limb-sparing surgery, although they can receive treatments such as radiofrequency ablation and radiation therapy. Doxorubicin hydrochloride(DOX), a first-line broad-spectrum antitumor drug, plays a crucial role in the treatment of osteosarcoma, but the efficacy of this drug is only 15-35% (5). Moreover, because DOX cannot target the tumor site after entering the body, it can cause adverse reactions in other organs (6).

In recent years, based on progress in tumor molecular biology research, the synergistic combination of chemical drugs with gene therapy has been widely studied (7, 8). Gene therapy is a promising treatment modality that achieves precision treatment by specifically targeting disease-related genes (9). With intensive research on the molecular mechanism of endogenous RNA interference, small interfering RNA (siRNAs) have become novel nucleic acid drugs for the treatment of diseases such as cancer (10). In 2018, the U.S. Food and Drug Administration (FDA) and the European Commission (EC) approved the commercial therapeutic drug ONPATTRO®(patisiran, ALNTTR02) for the treatment of peripheral nerve disease (polyneuropathy) caused by hereditary thyroid hormone-mediated amyloidosis (HATTR) in adults as the first commercial RNAi-based drug (9, 11). ONPATTRO^®^ is an siRNA targeting the transthyretin protein (TTR) wrapped in a lipid nanoparticle(NP) that acts to control this disease by silencing specific RNAs. siRNAs are short, synthesizable, double-stranded RNA molecules that have the ability to silence specific genes (12, 13). Although the therapeutic prospects of siRNAs are very promising, there are still some problems that prevent them from being widely used. The main reason is that siRNAs are large molecule drugs and are negatively charged, which makes them less likely to cross cell membranes. However, delivering siRNA *via* cationic liposomes also has its disadvantages such as a lack of targeting and rapid hepatic clearance (14). Determining how to accurately and effectively deliver siRNAs to their target sites has become a top priority for researchers. Given these challenges, a suitable nanocarrier is needed to transport chemotherapeutic drugs and siRNAs to tumor cells together to achieve synergistic therapeutic effects.

Poly (lactide-co-glycolide acid) (PLGA) is a biodegradable polymer with low toxicity, biocompatibility, and controlled release capability that is used to prepare NPs and has been approved by the U.S. Food and Drug Administration (FDA) for use in humans (15). Thus, PLGA NPs are a suitable drug delivery platform for carrying both drugs and siRNAs. Both PLGA and siRNA are negatively charged, which can lead to a decrease in siRNA encapsulation. This problem was resolved by adding positively charged polyethyleneimine (PEI) during the preparation process. However, PLGA NPs are usually captured and cleared by the reticuloendothelial system (RES) upon entry into the body; in addition, PLGA NPs are not targeted and therefore have a reduced ability to deliver drugs (16). In the past, researchers have had great success in modifying NPs with polyethylene glycols (PEG) to give them a “cloak of invisibility” to evade the immune clearance in the body (17). However, it has also been shown that PEG modifications can lead to problems such as immunogenicity and kidney damage, which can only be avoided by tedious synthetic methods, making the process of NP preparation complex and difficult (18). In recent years, researchers have developed new biomimetic NPs in which the core of the NP is covered with a natural cell membrane. This technique, which preserves the unique properties of the cell membrane, gives the NPs the unique functions of various cell types (19).

Mesenchymal stem cells (MSCs) are multifunctional cells with a unique regenerative capacity and immunomodulatory properties (20). MSCs possess unique tumor-homing and migration effects and can be used as drug carriers for the targeted treatment of tumors and metastatic diseases (21). These special characteristics of MSCs can be ascribed to the various receptors present on the cell membrane, including those for growth factors, cytokines, chemokines, and cell–matrix and cell−cell interactions (22). The CXCL12 that is released from tumor cells or at the site of tissue damage can interact with CXCR4 on the mesenchymal stem cell membrane (MSCM) to cause MSCs to accumulate at tumor sites (23, 24). However, some studies suggest that MSCs may also promote the pathogenesis of cancer or other diseases (25, 26). Using MSCMs as a “cloak” to camouflage NPs is safer than using MSCs themselves, as it reduces the risk associated with applying live proliferating cells while retaining their tumor-homing ability. Such camouflaged NPs can effectively evade clearance by the immune system, enhancing the therapeutic effect of the drug on the body (27, 28).

**Figure 1:**
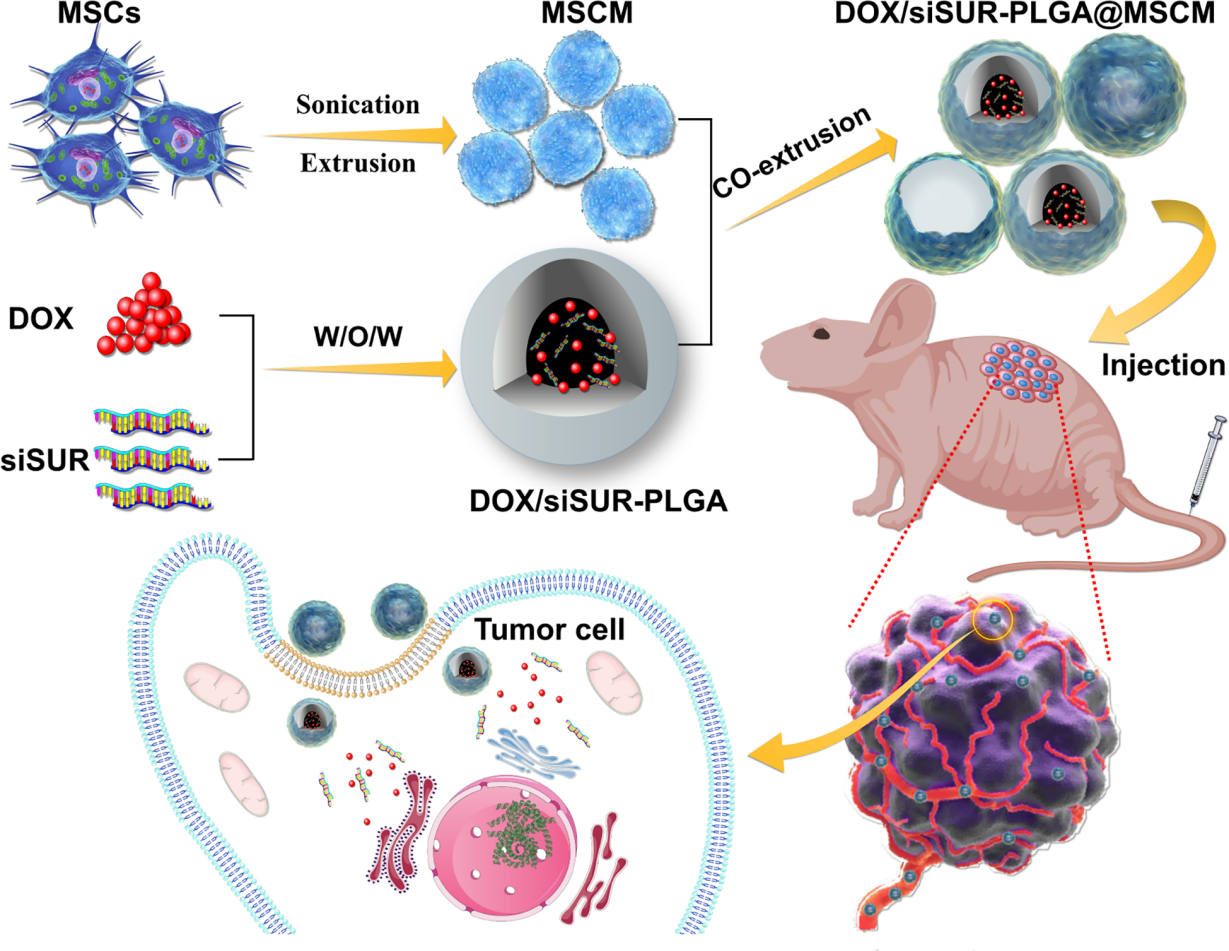
Mesenchymal stem cell membrane-camouflaged nanoparticles coloaded with DOX and survivin siRNA for osteosarcoma treatment.

Herein, we wrapped an siRNA targeting the apoptosis suppressor gene *survivin* and DOX in PLGA as a vehicle for drug delivery. Then, the NPs were covered with MSCMs to create DOX/siSUR-PLGA@MSCM NPs. These NPs take advantage of the immune escape ability, tumor-homing ability, and biocompatibility of MSCMs to enable better enrichment of NPs at the tumor site. The apoptosis suppressor gene *survivin* is a member of the family of inhibitory apoptosis proteins (IAPs) and is highly expressed in embryonic tissues and tumor cells (29, 30). siRNAs targeting this apoptosis suppressor gene inhibit the expression of *survivin* in tumor cells and promote the apoptosis of tumor cells, thus producing a synergistic effect with chemotherapeutic drugs (**Figure 1**).

## 2 Materials and methods

### 2.1 Materials

Poly (lactide-co-glycolide acid) (PLGA), with a lactide-glycolide ratio of 75:25 and molecular weight of 15 kDa, was purchased from Jinan Daigang Biomaterial Co., Ltd. (China). Polyvinyl alcohol (PVA, 13-23 kDa), PEI and DOX were purchased from Sigma Aldrich (USA). DiO and DiR were provided by Bestbio (China), and DAPI was obtained from Solarbio (China). siRNA sequences targeting human survivin (sense 5′-GCAAAGGAAACCAACAAUATT-3′, antisense 5′- UAUUGUUGGUUUCCUUUGCTT -3′), including FAM-labeled siRNAs, were made by Shanghai Gene Pharma (China). Anti-survivin (sc-17779) antibodies were obtained from Santa Cruz Biotechnology, and anti-GAPDH (ab8245) antibodies were purchased from Abcam (USA).

The MG63 human osteosarcoma cell line and the RAW264.7 mouse leukemia monocyte-macrophage cell line were purchased from the Cell Bank of the Chinese Academy of Sciences (Shanghai, China). High-glucose Dulbecco’s modified Eagle’s medium (DMEM), minimum essential medium (MEM), and fetal bovine serum (FBS) were purchased from Gibco (USA). The cells were cultivated in accordance with the ATCC’s suggested procedures.

BALB/c nude mice (4 weeks old) were purchased from the Vital River Company (Beijing, China). All animal experimental procedures were authorized by the Institutional Animal Care and Use Committee of Wish Company (Changchun, China) and followed the Guidelines for the Care and Use of Laboratory Animals of Jilin University, approval number 20220614-1.

### 2.2 Preparation of DOX/siSUR-PLGA NPs

DOX/siSUR-PLGA NPs were generated with the double-emulsion solvent evaporation technique according to a previously reported method with slight modifications (31). Briefly, 10 mg PLGA was dissolved in 1 ml DCM. Then, 66 µg of siRNA was mixed with 60 µg of PEI in RNase-free water and allowed to stand at room temperature for 20 min. Next, 0.6 mg DOX was dissolved in 100 µl RNase-free water and then added to the mixture of siSUR and PEI to produce an aqueous solution. The aqueous solution was added to the DCM solution mentioned above and emulsified with a probe sonicator (40% power and 5 s start/3 s pulse) in an ice bath for 2 min to obtain a primary W/O emulsion. Then, the primary emulsion was further emulsified with 4 mL 2% w/v PVA by sonication at 4°C for 10 min. After dilution using 20 mL of 1% PVA, the resulting product was stirred with a magnet for 3 hours to evaporate the DCM. The NPs were collected by centrifugation (Eppendorf, Germany) at 10000 × g for 10 min at 4°C and washed three times with RNAase-free deionized water to remove the PVA. Finally, the collected NPs were suspended and freeze-dried.

### 2.3 Extraction of mesenchymal stem cell membranes

Extraction of the mesenchymal stem cell membranes (MSCMs) was based on a method reported in a previous study (28). MSCs were digested, collected, resuspended in hypotonic buffer (1 mM NaHCO_3_, 1 mM PMSF, and 0.2 mM EDTA), and stored overnight at -80°C. The frozen aqueous solution was thawed at 4°C, and the solution was ultrasonicated with an ultrasonic probe for 3 min in an ice bath. The obtained liquid was centrifuged at 3200 × g for 10 min at 4°C, and the supernatant was collected. Finally, the supernatant was centrifuged at 20000 × g for 30 min at 4°C. The resultant precipitation product was considered to be the MSCMs. The protein concentration of the purified MSCMs was quantified using a BCA protein assay kit (Beyotime Biotech Corporation, Shanghai, China).

To prepare MSCM vesicles with an Avanti mini-extruder (Avanti Polar Lipids, Alabama, USA), the prepared membrane fragments were extruded through 400 and 200 nm porous polycarbonate membranes sequentially (20 times each).

### 2.4 Synthesis and characterization of DOX/siSUR-PLGA@MSCM NPs

DOX/siSUR-PLGA@MSCM NPs were synthesized with an Avanti mini-extruder by a previously reported method (32). An adequate amount of the prepared MSCM vesicle solution was mixed with the DOX/siSUR-PLGA NPs and extruded 15 times with a 200 nm polycarbonate porous membrane.

The hydrodynamic diameter of the NPs was measured by NP-tracking analysis using a NanoSight (NS300, Malvern Panalytical, Malvern, UK). Transmission electron microscopy (TEM, Hitachi H7650, Japan) and a Wyatt QELS instrument (DAWN EOS, Wyatt Technology, Goleta, CA, USA) were used to evaluate the NP morphology and zeta potential.

### 2.5 SDS-PAGE analysis of MSCM proteins

The samples were processed in SDS buffer, and the protein content of the produced MSCMs was analyzed using the BCA protein assay. The samples were separated *via* 10% SDS−PAGE at 100 V for 1.5 hours, and then proteins were stained with Coomassie Blue Super Fast Staining Solution (Beyotime Biotech Corporation, Shanghai, China) for 30 min, followed by destaining twice in heated water (33).

### 2.6 Encapsulation efficiency and drug-loading content

The encapsulation efficiency of DOX and siSUR was determined by collecting the supernatant during the preparation of the NPs. The concentrations of DOX and siSUR in the supernatant were determined by UV spectrophotometry and a microplate reader, respectively.

The drug encapsulation efficiency and drug-loading content were calculated using the following formulas:

Encapsulation efficiency (%) = Mass of drug loaded in NPs/Initial mass of drug × 100%

Loading content (%) = Mass of drug loaded in NPs/ mass of prepared NPs × 100%

### 2.7 *In vitro* release studies

The dialysis method was used to examine the release kinetics of DOX from PLGA NPs as previously reported. Briefly, equal amounts of DOX/siSUR-PLGA@MSCM NPs were suspended in dialysis bags containing 5 ml PBS buffer at pH 5 and 7.4. The dialysis bags were immersed in 100 mL PBS buffer with 1 M sodium salicylate with gentle shaking at 37°C, and then 1 ml of sample solution was collected at the indicated time points. The concentration of DOX was measured by UV−visible spectrophotometry, and 1 ml fresh PBS containing sodium salicylate was added.

### 2.8 *In vitro* cellular uptake study

To ensure that the MSCMs and the DOX/siSUR-PLGA core were colocalized, DiO was utilized to stain the MSCMs. MG63 cells were cultured on confocal plates overnight and subsequently incubated with DiO-labeled NPs for 3 hours. The cells were washed twice with PBS and fixed with 4% paraformaldehyde, and the nuclei were stained with DAPI. Observation was performed under a confocal laser scanning microscope (Olympus FV1000, Japan).

To evaluate the ability of the DOX/siSUR-PLGA@MSCM NPs to deliver both agents simultaneously, FAM-labeled siSUR and DOX were coloaded into the NPs. The subsequent steps were identical to those used in the colocalization assay described above.

MG63 and RAW264.7 cells were cultured in 6-well plates overnight, incubated with DOX/siSUR-PLGA or DOX/siSUR-PLGA@MSCM NPs for 3 hours, washed with PBS, stained with DAPI, and visualized with a fluorescence microscope (Nikon, Tokyo, Japan). Quantitative detection was performed by flow cytometry (Beckman Coulter, Brea, CA, USA).

### 2.9 Cell viability assay

A CCK-8 assay was used to evaluate the effect of the NPs on cell viability. Initially, 1 × 10^4^ MG63 cells were seeded per well in a 96-well culture plate and cultured for 24 hours. Subsequently, the cells were treated for 24 hours with Dox, DOX-PLGA NPs, DOX-PLGA@MSCM NPs, DOX/siSUR-PLGA NPs, or DOX/siSUR-PLGA@MSCM NPs with equivalent DOX concentrations of 0, 1, 2, 4 or 8 µM.

After washing the cells twice with PBS, the reagent was added according to the instructions of the CCK-8 kit (Beyotime Biotech Corporation, Shanghai, China), and the cells were incubated for 3 hours in an incubator at 37°C in the dark. To avoid the influence of DOX fluorescence on the experimental results, the supernatant was transferred to a new 96-well plate, and the absorbed light intensity at 450 nm was measured with a microplate reader.

### 2.10 Quantitative measurement of survivin gene silencing(qRT-PCR)

The silencing effect of NPs containing survivin siRNA was verified by qRT−PCR. MG63 cells were cultured overnight in 6-well plates, and 100 pmol survivin siRNA was transfected into MG63 cells with DOX/siSUR-PLGA@MSCM NPs and commercially available Lipo8000™ (Beyotime Biotech Corporation, Shanghai, China). After 24 hours of culture, total RNA was extracted from the cells with TRIzol (Thermo Fisher, USA) according to the manufacturer’s instructions. Then, cDNA was obtained with a Reverse PrimeScript RT reagent kit (Takara, Japan) according to the manufacturer’s instructions. Finally, qRT−PCR was performed with SYBR reagent (Roche). Amplification was performed with a QuantStudioTM Dx Real-Time instrument (Bio-Rad, USA). A real-time quantitative PCR instrument (Eppendorf, USA) was used for quantitative amplification. The ΔΔCt method was used to calculate the relative transcript level of survivin, and GAPDH was used as a reference gene for normalization.

### 2.11 Western blotting

MG63 cells were treated with the same method used in the previous qRT−PCR experiment. After 48 hours of culture, the total protein in the cells was extracted and quantified with a BCA kit. The extracted proteins were separated by SDS−PAGE and transferred to a 0.2 μm PVDF membrane (Millipore, USA). Subsequently, the PVDF membranes were blocked with 5% milk powder and incubated with primary antibodies at 4°C overnight. After washing with TBST 3 times, the fluorescent secondary antibody was added, and the samples were incubated for 1 hour. The expression of the target protein was detected with western blot equipment using GAPDH as a reference protein.

### 2.12 *In vivo* and ex vivo imaging of DOX/siSUR-PLGA@MSCM NPs

The subcutaneous tumor model was established by injecting MG63 cells (1×10^7^) into the right axilla of female BALB/C nude mice (4-6 weeks, 14-18 g). After injection of DiR-PLGA or DiR-PLGA@MSCM NPs, whole-body fluorescence images of the mice were captured at 8, 24, and 48 hours using a small animal imager (IVIS Spectrum, USA). Forty-eight hours after injection, the mice were sacrificed, and the major organs and tumors were collected for *in vitro* imaging.

### 2.13 *In vivo* tumor studies and biosafety

A nude mouse model bearing MG63 tumors was established using the previously described method, and tumors were allowed to grow to 100 mm^3^ in size. All nude mice were randomly divided into 5 groups. The mice were treated for 14 days with PBS, DOX, DOX-PLGA NPs, DOX/siSUR-PLGA NPs, and DOX/siSUR-PLGA@MSCM NPs by tail vein injection every 2 days. The concentration of DOX was fixed at 5 mg/kg.

During treatment, changes in tumor volume and relative body weight were monitored. Tumor volume was calculated according to the formula volume= (short diameter^2^×long longitude)/2. After the last treatment, the mice were observed for 4 days and sacrificed, and the tumors were collected for H&E staining, immunohistochemistry, and TUNEL analysis. In parallel, the major organs were collected for H&E staining to assess the biosafety of the NPs. The tumor and major organ tissues were placed in 4% paraformaldehyde and fixed overnight and then routinely processed into 4 μm paraffin sections, dewaxed, stained with hematoxylin, dehydrated in gradient alcohol, and stained with eosin. After antigen repair, the tumor tissue sections were treated with anti-survivin antibody (sc-17779, Santa Cruz Biotechnology) and hematoxylin was added to re-stain the cell nuclei before observation under a light microscope. Tumor tissue sections were also dewaxed and stained with the reagents from a TUNEL assay kit (Beyotime Biotech Corporation, Shanghai, China) and the nuclei were restained with DAPI before observation under a fluorescence microscope (Olympus, Japan).

### 2.14 Statistical analysis

Experiments were performed in triplicate.

The data were analyzed with GraphPad Prism 9 software (La Jolla, CA, USA). Statistical evaluations of data were performed using Student’s t test or two-way analysis of variance (ANOVA). All results are expressed as the mean ± standard deviation (SD).

A p value <0.05 was considered statistically significant.

## 3 Results and discussion

### 3.1 Preparation and characterization of DOX/siSUR-PLGA@MSCM NPs

In the process of preparing DOX/siSUR-PLGA NPs by the emulsion volatilization method, PEI was added to more effectively bind the negatively charged siRNA to improve the siRNA drug loading rate (34). The obtained NPs were then sonicated and treated to enable effective binding to the mesenchymal stem cell membrane. DOX-PLGA, siSUR-PLGA, and DOX-PLGA@MSCM NPs were prepared using the method described above.

**Figure 2:**
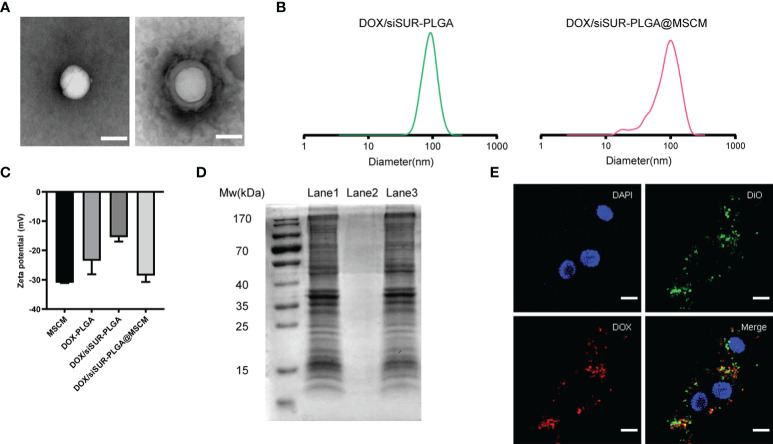
Characterization of the nanoparticles. **(A)** TEM images of DOX/siSUR-PLGA NPs (left) and DOX/siSUR-PLGA@MSCM NPs (right). Scale bar, 100 nm. **(B)** Size of DOX/siSUR-PLGA NPs (left, green) and DOX/siSUR-PLGA@MSCM NPs (right, red). **(C)** Zeta potentials of the prepared nanoparticles. **(D)** Analysis of the proteins in the lysates of MSC membranes (Lane 1), DOX/siSUR-PLGA NPs (Lane 2) and DOX/siSUR-PLGA@MSCM NPs (Lane 3) by SDS−PAGE. **(E)** Intracellular colocalization of MSC membranes (DiO, green) and DOX/siSUR-PLGA NPs (DOX, red). MG63 cell nuclei were stained with DAPI (blue). Scale bar, 20 μm.

The structures of DOX/siSUR-PLGA and DOX/siSUR-PLGA@MSCM NPs were observed using transmission electron microscopy. Both types of NPs were spherical in shape, and a cell membrane covering the surface of DOX/siSUR-PLGA@MSCM NPs could be observed (**Figure 2A**). This indicates that NPs completely encapsulated by MSCMs can be obtained by the above method. We also examined the particle size of the NPs, and NanoSight measurements showed that the particle size of the NPs encapsulated by cell membranes increased by approximately 16 nm (**Figure 2B**). Meanwhile, the zeta sizer data showed that the surface charge of the NPs covered by MSCMs changed from -15.4 mV to -28.6 mV, which further confirmed that the NPs were covered by the cell membrane (**Figure 2C**). In addition, we measured the encapsulation rate and drug-loading rate of DOX by UV spectrophotometry at 480 nm, and the obtained values were 32.34 ± 2.19% and 3.98 ± 0.24%, respectively. The content of siRNA in the NPs was determined by measuring the content of ribonucleotide in the supernatant, and the encapsulation rate and drug-loading rate were 53.10 ± 1.45% and 53.94 ± 2.31 (μg/10mg), respectively (**Table S1**).

It is well known that membrane protein coated NPs have to retain their biological characteristics to maintain their biomimetic functions. Therefore, we verified whether the proteins of the MSCM were successfully transferred to the NPs by protein gel electrophoresis and Coomassie Brilliant Blue staining. The protein bands of the MSCM and DOX/siSUR-PLGA@MSCM lysis products were almost identical, while the bare NPs showed no protein bands. This indicates that the method used in this paper can preserve the protein properties of cell membranes and is not affected by the storage of the particles (**Figure 2D**).

We used DOX self-fluorescence (red) and DiO (green)-labeled MSCMs to observe the core-shell structure of the NPs after uptake by cells. The labeled NPs were coincubated with MG63 cells in an incubator for 3 hours. The red fluorescence of DOX and the fluorescence distribution of DiO-labeled MSCMs overlapped when observed by laser confocal microscopy. This strongly indicates that the NPs that were taken up by the tumor cells were still able to retain an intact structure inside the cells (**Figure 2E**).

### 3.2 Drug release profile

**Figure 3:**
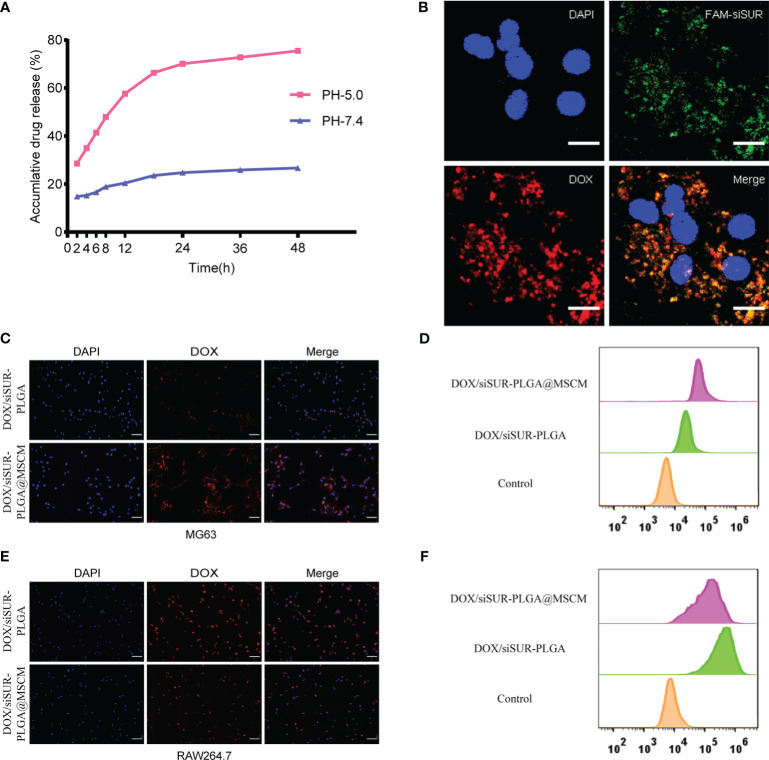
*In vitro* validation of the *in vitro* release effect, cotransport ability, tumor-homing property and immune escape ability of DOX/siSUR-PLGA@MSCM nanoparticles. **(A)** *In vitro* release of DOX at pH 5.0 and pH 7.4. **(B)** CLSM image of the DOX/siSUR-PLGA@MSCM nanoparticles after being taken up by MG63 cells. DOX self-fluorescence (red), FAM-labeled siSUR (green), and MG63 cell nuclei stained with DAPI (blue). Scale bar, 20 μm. **(C)** MG63 cells were treated with DOX/siSUR-PLGA and DOX/siSUR-PLGA@MSCM NPs, and phagocytosis was observed by fluorescence microscopy using the fluorescence of DOX (red); nuclei were stained with DAPI. Scale bar, 100 μm. **(D)** MG63 cells were cocultured with nanoparticles for 3 hours, and then nanoparticle phagocytosis was detected by flow cytometry. **(E)** RAW264.7 cells were treated with DOX/siSUR-PLGA NPs and DOX/siSUR-PLGA@MSCM NPs and phagocytosis was observed by fluorescence microscopy using the fluorescence of DOX (red); nuclei were stained with DAPI. Scale bar, 100 μm. **(F)** RAW264.7 cells were cocultured with nanoparticles for 3 hours, and then nanoparticle was detected by flow cytometry.

Subsequently, we investigated drug release from the DOX/siSUR-PLGA@MSCM NPs *in vitro*. Under conditions simulating the biological environment *in vivo*, the NPs were allowed to undergo drug release in PBS buffer with pH= 7.4 and pH= 5, and the release curves were analyzed (**Figure 3A**). When the pH was 7.4, the cumulative release of DOX from the DOX/siSUR-PLGA@MSCM NPs over 48 hours was 26.67%; however, the cumulative release of DOX at pH 5 was 75.52%. One common feature of cancer is altered glucose metabolism and increased glycolysis. In contrast to normal cells, which are predominantly aerobic, tumor cells depend more on anaerobic glycolysis, and the resulting metabolite lactate acidifies the tumor microenvironment (35, 36). These results showed that an acidic environment was more favorable for the release of the drugs from the NPs.

### 3.3 Cellular uptake

To ensure that the prepared NPs have the ability to codeliver DOX and siSUR, we labeled siRNA with FAM and loaded it onto the NPs using the drug-loading method described above. Colocalization of siSUR and DOX in MG63 cells was observed by laser confocal microscopy (**Figure 3B**).

The chemotactic factor CXCR4 gives the MSCM a clear tumor-targeting property, which can increase the accumulation of NPs at the tumor site. For this reason, we observed the cellular internalization behavior of DOX/siSUR-PLGA@MSCM and DOX/siSUR-PLGA NPs coincubated with MG63 cells for 3 hours. Under the fluorescence microscope (**Figure 3C**), the fluorescence intensity of the NPs disguised by the MSCM was stronger in MG63 cells. This suggests that more DOX/siSUR-PLGA@MSCM NPs are taken up by MG63 cells, a result that was confirmed by flow cytometry analysis (**Figure 3D**).

Subsequently, we examined the uptake of DOX/siSUR-PLGA@MSCM NPs and DOX/siSUR-PLGA NPs by RAW264.7 mouse macrophages after 3 hours by fluorescence microscopy and flow cytometric analyses. Fluorescence microscopy (**Figure 3E**) revealed that the NPs camouflaged with MSCMs displayed lower fluorescence intensity and were less engulfed by RAW264.7 cells, a trend that was verified by flow cytometry (**Figure 3F**). This suggests that NPs camouflaged by MSCMs have better biocompatibility. As exogenous drugs, NPs are easily recognized and phagocytosed by macrophages in the human body. However, the MSCM can disguise the NPs, enabling their escape or delaying macrophage phagocytosis, ultimately increasing their cycle time in the body while maintaining drug stability.

### 3.4 *In vitro* antitumor and gene-silencing effects of DOX/siSUR-PLGA@MSCM NPs

**Figure 4:**
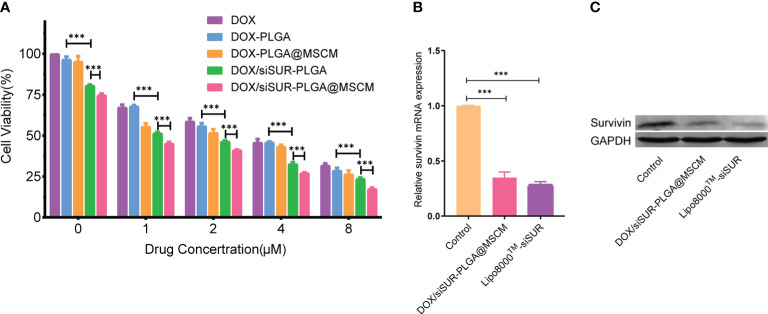
Evaluation of the cytotoxicity and gene-silencing efficiency of the nanoparticles. **(A)** Viability of MG63 cells after treatment with various nanoparticles containing DOX at different concentrations. MG63 cells were transfected with siRNA using Lipo8000™ or treated with DOX/siSUR-PLGA@MSCM nanoparticles containing the same concentration of siRNA, and the gene and protein expression of *survivin* was determined by qRT−PCR **(B)** and western blotting **(C)**, respectively. ***p <0.001, n=3.

We evaluated the toxic effects of free DOX, DOX-PLGA NPs, DOX-PLGA@MSCM NPs, DOX/siSUR-PLGA NPs, and DOX/siSUR-PLGA@MSCM NPs on MG63 cells (**Figure 4A**). The results showed that DOX-PLGA had a similar killing effect on tumor cells compared with different concentrations of free DOX. Meanwhile, the DOX-PLGA@MSCM NPs were more toxic than the treatments in the first two groups, probably due to the greater uptake of the MSCM-modified NPs. In addition, the two groups loaded with siRNA more effectively killed tumor cells because survivin siRNA loaded in the NPs promoted the apoptosis of tumor cells. Of course, the DOX/siSUR-PLGA@MSCM NPs had the strongest killing effect on tumor cells due to both the gene-silencing effect of siSUR and the camouflage effect of the MSCMs.

To verify the gene-silencing effect of survivin siRNA-loaded NPs, we examined the expression levels of survivin mRNA in transfected MG63 cells by quantitative reverse transcription-polymerase chain reaction (qRT−PCR). Using commercial Lipo8000™ as the transfection reagent, after comparison with cells treated with DOX/siSUR-PLGA@MSCM NPs, the experimental results confirmed that both methods resulted in downregulated expression of survivin mRNA and similar effects on gene silencing (**Figure 4B**). Moreover, the immunoblotting results showed that the survivin protein level was significantly reduced in MG63 cells after DOX/siSUR-PLGA@MSCM NP treatment, which further verified the gene silencing effect of siRNA-loaded NPs (**Figure 4C**).

### 3.5 Biodistribution *in vivo* and *in vitro*

To investigate whether MSCM-camouflaged NPs remain targeted and escape the immune system in the organism, we used a small animal live imaging system to study the *in vivo* biodistribution of naked NPs and cell membrane-camouflaged NPs in MG63 tumor-bearing mice.

**Figure 5:**
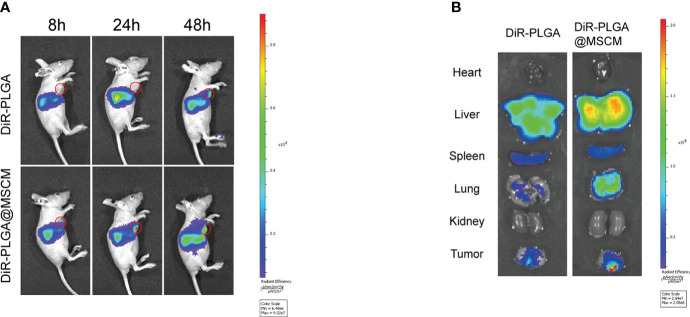
Biodistribution of the nanoparticles *in vivo* and *in vitro*. **(A)** Whole-body fluorescence images of mice with subcutaneous MG63 xenograft tumors at 8, 24 and 48 hours after DiR-PLGA and DiR-PLGA@MSCM NP injection. **(B)** *In vitro* images of the tumor, heart, liver, spleen, spleen, lung and kidney at 48 hours after injection of DiR-PLGA and DiR-PLGA@MSCM NPs.

The infrared light emitted by the fluorescent probe DiR can effectively penetrate cells and tissues and is widely used for *in vivo* distribution studies of NPs. We prepared DiR-PLGA and DiR-PLGA@MSCM NPs using DiR as a tracer and injected them *via* the tail vein into BALB/c nude mice with homogeneous MG63 tumors. As shown in **Figure 5A**, the fluorescence intensity at the tumor site 48 hours after intravenous injection was weaker in nude mice injected with DiR-PLGA NPs than in nude mice injected with DiR-PLGA@MSCM NPs. These results suggest that NPs camouflaged with MSCMs can target tumor sites. In contrast, bare NPs are slightly less effective in targeting tumor sites due to the immune system and metabolism by the liver and kidneys. The effect of the *in vivo* treatment is consistent with the results obtained from the *in vitro* studies. Subsequently, we performed a NP distribution study in mouse organ tissues by sacrificing mice 48 hours after injection and collecting tumors and major organs for *in vitro* imaging. The *in vitro* fluorescence images (**Figure 5B**) showed that the fluorescence intensity of DiR-PLGA@MSCM NPs in tumors was significantly higher than that of DiR-PLGA NPs, indirectly confirming that MSCMs have tumor-homing ability. We also found that the fluorescence intensity of DiR-PLGA NPs in the liver and lung was slightly lower than that of DiR-PLGA@MSCM NPs, which may be due to the absence of the camouflage MSCMs and the rapid clearance of DiR-PLGA NPs by the immune system. In addition, the fluorescent probe DiR binds to lipophilic biomolecules *in vivo* after its release, resulting in enhanced fluorescence intensity. Thus, DiR-PLGA@MSCM NPs have better biocompatibility and release more DiR.

### 3.6 *In vivo* antitumor efficacy

To verify that the prepared NPs had good antitumor effects, we evaluated the therapeutic effects of different DOX-containing NPs on MG63 tumor-bearing nude mice.

**Figure 6:**
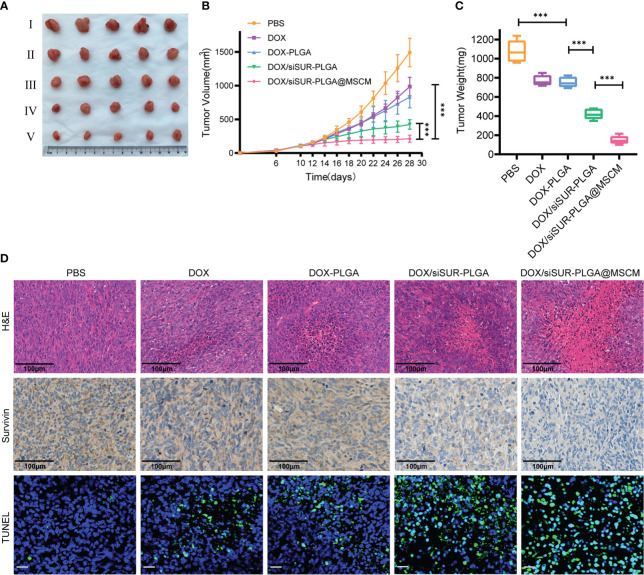
*In vivo* treatment of tumors. **(A)** Subcutaneous tumor-bearing MG63 mice were treated at a fixed DOX dose (5 mg/kg) every 2 days by intravenous injection. Start treatment on day 10, end treatment on day 24, and after 8 treatments observation for 4 days, photographs of tumor tissues were captured on day 28 (I PBS, II DOX, III DOX-PLGA, IV DOX/siSUR-PLGA NPs, V DOX/siSUR-PLGA@MSCM NPs). Tumor growth during treatment **(B)** and tumor weight after treatment **(C)** were recorded. **(D)** H&E staining of tumor tissue (scale bar, 100 μm), immunohistochemical analysis of *survivin* expression (scale bar, 100 μm), and TUNEL assay data (scale bar, 25 μm). ***p <0.001, n=5.

In this study, mice were given tail vein injections of DOX at a dose of 5 mg/kg. Based on these treatments, DOX, DOX-PLGA NPs, DOX/siSUR-PLGA NPs, and DOX/siSUR-PLGA@MSCM NPs all exerted tumor-suppressive effects. Although DOX is a commercial agent commonly used in clinical practice, its therapeutic effect is very limited when used alone. Even with PLGA-modified DOX in the DOX-PLGA treatment group, its specific targeting ability is poor, its half-life is short, it is easily cleared by the reticuloendothelial system *in vivo*, and its therapeutic effect is limited. However, once DOX was coloaded with survivin siRNA in the NPs, the antitumor effect was improved, which may be inextricably related to the synergistic effect of the two agents. As seen from the results (**Figures 6A, B, C**), after treatment, the DOX/siSUR-PLGA@MSCM group had the most pronounced tumor suppression and the smallest tumor volume and weight among the groups.

In addition, we performed H&E staining of the removed tumors (**Figure 6D**), and we observed that the tumors in the DOX/siSUR-PLGA@MSCM group had the largest necrotic area, which also confirmed the advantage of the DOX/siSUR-PLGA@MSCM treatment compared with the other treatments. This is because DOX/siSUR-PLGA@MSCM NPs delivered two agents together, exploiting the synergistic effect of the agents, while also enhancing the targeting of the NPs *in vivo* through the camouflage effect of MSCMs and avoiding immune clearance.

We also verified the gene silencing effect of siRNA by immunohistochemistry. The survivin expression level was not significantly reduced in the DOX and DOX-PLGA groups (**Figure 6D**). Additionally, the silencing of this apoptosis-suppressing gene was more obvious in the DOX/siSUR-PLGA and DOX/siSUR-PLGA@MSCM groups where the MSCM pseudo-NPs had the best effect.

Subsequently, we performed fluorescence analyses of the tumor tissues from each group. The green fluorescence in **Figure 6D** represents apoptotic cells, and the tumor cells in each treatment group had different degrees of apoptosis. Among them, the amount of apoptotic tumor cells in the DOX and DOX-PLGA groups was approximately the same while the other two treatments better promoted apoptosis, among which the DOX/siSUR-PLGA@MSCM NPs produced the best effects. The above results showed more pronounced tumor suppression and accelerated apoptosis of tumor cells based on the synergistic effect of the two drugs.

### 3.7 Biosafety evaluation

**Figure 7:**
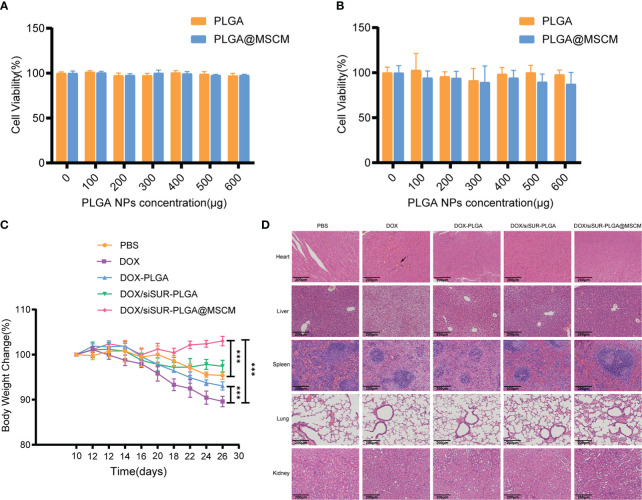
*In vivo* and *in vitro* biosafety assessment. Cytotoxicity of different concentrations of empty PLGA and empty PLGA@MSCM nanoparticles to MG63 cells, assessed at 24 hours **(A)** and 48 hours **(B)**. **(C)** Changes in body weight in the mice in each group during treatment. **(D)** H&E staining images of the major organs of mice from different treatment groups. Scale bar, 100 µm. The arrow points to a myocardial vascular lesion. ***p <0.001.

Biosafety is an important consideration for the clinical application of NPs. To examine this issue, we evaluated the toxicity of the prepared NPs in cells by a CCK8 assay. Empty PLGA microspheres and MSCM-coated PLGA microspheres were cocultured with MG63 cells at different concentrations for 24 (**Figure 7A**) and 48 hours (**Figure 7B**), and the cell survival rate was above 80%. Throughout the treatment, we recorded the changes in the body weight of the mice, and we found that the mice in the DOX injection group had the most significant weight loss (**Figure 7C**), which may be due to the significant myelosuppression and cardiotoxicity of DOX. In contrast, in the DOX/siSUR-PLGA@MSCM treatment group, the mice had less fluctuation in body weight, indicating a reduction in the toxic effects of the drug, but still showed inhibition of tumor growth.

Subsequently, we performed H&E staining of the visceral tissues of the mice at the end of treatment. In the prepared tissue sections, thickened myocardial vessels with collagenized changes were observed in the mice from the DOX group, which could lead to myocardial ischemia and cause cardiac necrosis. The mice in this group also had obvious ballooning-type changes in the liver cells, which is a sign of chronic liver injury. In contrast, there were no obvious pathological changes in the organs of the other groups. (**Figure 7D**). These results suggest that the NPs we prepared and applied have better biocompatibility and biosafety than conventional chemotherapeutic drugs and that they can reduce the biological toxicity of chemotherapeutic drugs.

## 4 Conclusion

In this study, biomimetic NPs camouflaged by MSCMs were successfully prepared by coloading PLGA NPs with the conventional chemotherapeutic agents DOX and siSUR. The stability, targeting ability, and immune escape ability of these NPs were utilized to induce apoptosis of osteosarcoma cells through the synergistic effects of the two agents, which provide a precise combined treatment for osteosarcoma cells *in vivo* and *in vitro*. DOX/siSUR-PLGA@MSCM NPs showed significant inhibition of tumor growth in both *in vivo* and *in vitro* experiments and reduced the toxicity of the free chemotherapeutic drugs. These findings provide a delivery strategy for targeted therapy and a combination of drugs for treatment of osteosarcoma. This strategy can also be used to develop other codelivery systems for antitumor drugs and siRNAs.

## Data availability statement

The original contributions presented in the study are included in the article/supplementary material. Further inquiries can be directed to the corresponding authors.

## Ethics statement

All animal experimental procedures were authorized by the Institutional Animal Care and Use Committee of Wish Company (Changchun, China) and followed the Guidelines for the Care and Use of Laboratory Animals of Jilin University.

## Author contributions

JZ designed the study and performed both the *in vitro* and biological experiments. XM participated in designing and supervising the experiments. JZ, XH and XZ performed animal modeling and the animal experiments. XZ assisted with the biological experiments. JZ wrote the manuscript. XH and XZ assisted in visualizing the data. and PL and JJ supervised and directed the entire body of work and revised the paper. All authors contributed to the article and approved the submitted version.

## References

[1] Picci P. Osteosarcoma (Osteogenic sarcoma). Orphanet J Rare Dis (2007) 2:6. doi: 10.1186/1750-1172-2-6 17244349 PMC1794406

[2] Bielack SS Kempf-Bielack B Winkler K. Osteosarcoma: Relationship of response to preoperative chemotherapy and type of surgery to local recurrence. J Clin Oncol (1996) 14(2):683–4. doi: 10.1200/jco.1996.14.2.683 8636791

[3] Bielack SS Kempf-Bielack B Delling G Exner GU Flege S Helmke K. Prognostic factors in high-grade osteosarcoma of the extremities or trunk: An analysis of 1,702 patients treated on neoadjuvant cooperative osteosarcoma study group protocols. J Clin Oncol (2002) 20(3):776–90. doi: 10.1200/jco.2002.20.3.776 11821461

[4] Wittig JC Bickels J Priebat D Jelinek J Kellar-Graney K Shmookler B. Osteosarcoma: A multidisciplinary approach to diagnosis and treatment. Am Family physician (2002) 65(6):1123–32. 11925089

[5] Antman K Crowley J Balcerzak SP Rivkin SE Weiss GR Elias A. An intergroup phase iii randomized study of doxorubicin and dacarbazine with or without ifosfamide and mesna in advanced soft tissue and bone sarcomas. J Clin Oncol (1993) 11(7):1276–85. doi: 10.1200/jco.1993.11.7.1276 8315425

[6] Carvalho C Santos RX Cardoso S Correia S Oliveira PJ Santos MS. Doxorubicin: The good, the bad and the ugly effect. Curr Med Chem (2009) 16(25):3267–85. doi: 10.2174/092986709788803312 19548866

[7] Luo K Gao Y Yin S Yao Y Yu H Wang G. Co-Delivery of paclitaxel and Stat3 sirna by a multifunctional nanocomplex for targeted treatment of metastatic breast cancer. Acta Biomater (2021) 134:649–63. doi: 10.1016/j.actbio.2021.07.029 34289420

[8] Dai X Tan C. Combination of microrna therapeutics with small-molecule anticancer drugs: Mechanism of action and Co-delivery nanocarriers. Adv Drug Delivery Rev (2015) 81:184–97. doi: 10.1016/j.addr.2014.09.010 25281917

[9] Hu B Zhong L Weng Y Peng L Huang Y Zhao Y. Therapeutic sirna: State of the art. Signal Transduct Target Ther (2020) 5(1):101. doi: 10.1038/s41392-020-0207-x 32561705 PMC7305320

[10] Oh YK Park TG. Sirna delivery systems for cancer treatment. Adv Drug Delivery Rev (2009) 61(10):850–62. doi: 10.1016/j.addr.2009.04.018 19422869

[11] Weng Y Xiao H Zhang J Liang XJ Huang Y. Rnai therapeutic and its innovative biotechnological evolution. Biotechnol Adv (2019) 37(5):801–25. doi: 10.1016/j.biotechadv.2019.04.012 31034960

[12] Zhuang J Gong H Zhou J Zhang Q Gao W Fang RH. Targeted gene silencing *in vivo* by platelet membrane-coated metal-organic framework nanoparticles. Sci Adv (2020) 6(13):eaaz6108. doi: 10.1126/sciadv.aaz6108 32258408 PMC7101224

[13] McManus MT Sharp PA. Gene silencing in mammals by small interfering rnas. Nat Rev Genet (2002) 3(10):737–47. doi: 10.1038/nrg908 12360232

[14] Aagaard L Rossi JJ. Rnai therapeutics: Principles, prospects and challenges. Adv Drug Delivery Rev (2007) 59(2-3):75–86. doi: 10.1016/j.addr.2007.03.005 PMC1978219 17449137

[15] Ravi Kumar MN Bakowsky U Lehr CM. Preparation and characterization of cationic plga nanospheres as DNA carriers. Biomaterials (2004) 25(10):1771–7. doi: 10.1016/j.biomaterials.2003.08.069 14738840

[16] Gao X Li L Cai X Huang Q Xiao J Cheng Y. Targeting nanoparticles for diagnosis and therapy of bone tumors: Opportunities and challenges. Biomaterials (2021) 265:120404. doi: 10.1016/j.biomaterials.2020.120404 32987273

[17] Hu CM Zhang L Aryal S Cheung C Fang RH Zhang L. Erythrocyte membrane-camouflaged polymeric nanoparticles as a biomimetic delivery platform. Proc Natl Acad Sci USA (2011) 108(27):10980–5. doi: 10.1073/pnas.1106634108 PMC3131364 21690347

[18] Harris JM Chess RB. Effect of pegylation on pharmaceuticals. Nat Rev Drug Discovery (2003) 2(3):214–21. doi: 10.1038/nrd1033 12612647

[19] Fang RH Kroll AV Gao W Zhang L. Cell membrane coating nanotechnology. Adv Mater (2018) 30(23):e1706759. doi: 10.1002/adma.201706759 29582476 PMC5984176

[20] Chen FM Liu X. Advancing biomaterials of human origin for tissue engineering. Prog Polym Sci (2016) 53:86–168. doi: 10.1016/j.progpolymsci.2015.02.004 27022202 PMC4808059

[21] Gao Z Zhang L Hu J Sun Y. Mesenchymal stem cells: A potential targeted-delivery vehicle for anti-cancer drug, loaded nanoparticles. Nanomedicine (2013) 9(2):174–84. doi: 10.1016/j.nano.2012.06.003 22772046

[22] Wang M Xin Y Cao H Li W Hua Y Webster TJ. Recent advances in mesenchymal stem cell membrane-coated nanoparticles for enhanced drug delivery. Biomater Sci (2021) 9(4):1088–103. doi: 10.1039/d0bm01164a 33332490

[23] Cheng Z Ou L Zhou X Li F Jia X Zhang Y. Targeted migration of mesenchymal stem cells modified with Cxcr4 gene to infarcted myocardium improves cardiac performance. Mol Ther (2008) 16(3):571–9. doi: 10.1038/sj.mt.6300374 18253156

[24] Brenner S Whiting-Theobald N Kawai T Linton GF Rudikoff AG Choi U. Cxcr4-transgene expression significantly improves marrow engraftment of cultured hematopoietic stem cells. Stem Cells (2004) 22(7):1128–33. doi: 10.1634/stemcells.2003-0196 15579633

[25] Roorda BD ter Elst A Kamps WA de Bont ES. Bone marrow-derived cells and tumor growth: Contribution of bone marrow-derived cells to tumor micro-environments with special focus on mesenchymal stem cells. Crit Rev oncology/hematology (2009) 69(3):187–98. doi: 10.1016/j.critrevonc.2008.06.004 18675551

[26] Tsukamoto S Honoki K Fujii H Tohma Y Kido A Mori T. Mesenchymal stem cells promote tumor engraftment and metastatic colonization in rat osteosarcoma model. Int J Oncol (2012) 40(1):163–9. doi: 10.3892/ijo.2011.1220 21971610

[27] Kaneti L Bronshtein T Malkah Dayan N Kovregina I Letko Khait N Lupu-Haber Y. Nanoghosts as a novel natural nonviral gene delivery platform safely targeting multiple cancers. Nano Lett (2016) 16(3):1574–82. doi: 10.1021/acs.nanolett.5b04237 26901695

[28] Gao C Lin Z Jurado-Sanchez B Lin X Wu Z He Q. Stem cell membrane-coated nanogels for highly efficient *in vivo* tumor targeted drug delivery. Small (2016) 12(30):4056–62. doi: 10.1002/smll.201600624 27337109

[29] Ambrosini G Adida C Altieri DC. A novel anti-apoptosis gene, survivin, expressed in cancer and lymphoma. Nat Med (1997) 3(8):917–21. doi: 10.1038/nm0897-917 9256286

[30] Lin TY Chan HH Chen SH Sarvagalla S Chen PS Coumar MS. Birc5/Survivin is a novel Atg12-Atg5 conjugate interactor and an autophagy-induced DNA damage suppressor in human cancer and mouse embryonic fibroblast cells. Autophagy (2020) 16(7):1296–313. doi: 10.1080/15548627.2019.1671643 PMC7469615 31612776

[31] Xu C Liu W Hu Y Li W Di W. Bioinspired tumor-homing nanoplatform for Co-delivery of paclitaxel and sirna-E7 to hpv-related cervical malignancies for synergistic therapy. Theranostics (2020) 10(7):3325–39. doi: 10.7150/thno.41228 PMC7053183 32194871

[32] Mu X Li J Yan S Zhang H Zhang W Zhang F. Sirna delivery with stem cell membrane-coated magnetic nanoparticles for imaging-guided photothermal therapy and gene therapy. ACS Biomaterials Sci Eng (2018) 4(11):3895–905. doi: 10.1021/acsbiomaterials.8b00858 33429596

[33] Reisinger V Eichacker LA. Analysis of membrane protein complexes by blue native page. Proteomics (2006) 6(Suppl 2):6–15. doi: 10.1002/pmic.200600553 17031799

[34] Patil Y Panyam J. Polymeric nanoparticles for sirna delivery and gene silencing. Int J Pharm (2009) 367(1-2):195–203. doi: 10.1016/j.ijpharm.2008.09.039 18940242 PMC2660441

[35] Gatenby RA Gillies RJ. Why do cancers have high aerobic glycolysis? Nat Rev Cancer (2004) 4(11):891–9. doi: 10.1038/nrc1478 15516961

[36] Dang CV Lewis BC Dolde C Dang G Shim H. Oncogenes in tumor metabolism, tumorigenesis, and apoptosis. J bioenergetics biomembranes (1997) 29(4):345–54. doi: 10.1023/a:1022446730452 9387095

