# Supplementary material for: Synergistic treatment of osteosarcoma with biomimetic nanoparticles transporting doxorubicin and siRNA
Source: Front Oncol. 2023 Jan 23;13:1111855. doi: 10.3389/fonc.2023.1111855 (PMC9900173; doi:10.3389/fonc.2023.1111855)
Supplement: Supplementary file 1 [file Table_1.docx]

Supplementary Material

# Supplementary Table

**Table S1.** Encapsulation efficiency and drug loading content of various nanoparticle formulations.

| Samples | Encapsulation efficiency of DOX(%) | Encapsulation efficiency of siRNA(%) | Drug loading of DOX(%) | Drug loading of siRNA(μg/10mg) |
| --- | --- | --- | --- | --- |
| DOX-PLGA | 35.19±4.31% | - | 4.08±0.48% | - |
| siRNA-PLGA | - | 56.24±2.15% | - | 58.08±2.49 |
| DOX/siRNA-PLGA | 32.34±2.19% | 53.10±1.45% | 3.98±0.24% | 53.94±2.31 |

Data are shown as mean ± SD (n=3).
